# Changes in the geometry and robustness of diffusion tensor imaging networks: Secondary analysis from a randomized controlled trial of young autistic children receiving an umbilical cord blood infusion

**DOI:** 10.3389/fpsyt.2022.1026279

**Published:** 2022-10-20

**Authors:** Anish K. Simhal, Kimberly L. H. Carpenter, Joanne Kurtzberg, Allen Song, Allen Tannenbaum, Lijia Zhang, Guillermo Sapiro, Geraldine Dawson

**Affiliations:** ^1^Department of Medical Physics, Memorial Sloan Kettering Cancer Center, New York, NY, United States; ^2^Duke Center for Autism and Brain Development, Department of Psychiatry and Behavioral Sciences, Duke University School of Medicine, Durham, NC, United States; ^3^Marcus Center for Cellular Cures, Duke University Medical Center, Durham, NC, United States; ^4^Brain Imaging and Analysis Center, Duke University, Durham, NC, United States; ^5^Department of Computer Science, Stony Brook University, Stony Brook, NY, United States; ^6^Department of Applied Mathematics and Statistics, Stony Brook University, Stony Brook, NY, United States; ^7^Department of Electrical and Computer Engineering, Duke University, Durham, NC, United States; ^8^Department of Biomedical Engineering, Computer Science, and Mathematics, Duke University, Durham, NC, United States

**Keywords:** white matter, diffusion tensor imaging, clinical trial, biomarkers, stem cells

## Abstract

Diffusion tensor imaging (DTI) has been used as an outcome measure in clinical trials for several psychiatric disorders but has rarely been explored in autism clinical trials. This is despite a large body of research suggesting altered white matter structure in autistic individuals. The current study is a secondary analysis of changes in white matter connectivity from a double-blind placebo-control trial of a single intravenous cord blood infusion in 2–7-year-old autistic children (1). Both clinical assessments and DTI were collected at baseline and 6 months after infusion. This study used two measures of white matter connectivity: change in node-to-node connectivity as measured through DTI streamlines and a novel measure of feedback network connectivity, Ollivier-Ricci curvature (ORC). ORC is a network measure which considers both local and global connectivity to assess the robustness of any given pathway. Using both the streamline and ORC analyses, we found reorganization of white matter pathways in predominantly frontal and temporal brain networks in autistic children who received umbilical cord blood treatment versus those who received a placebo. By looking at changes in network robustness, this study examined not only the direct, physical changes in connectivity, but changes with respect to the whole brain network. Together, these results suggest the use of DTI and ORC should be further explored as a potential biomarker in future autism clinical trials. These results, however, should not be interpreted as evidence for the efficacy of cord blood for improving clinical outcomes in autism. This paper presents a secondary analysis using data from a clinical trial that was prospectively registered with ClinicalTrials.gov(NCT02847182).

## Introduction

Previous research has demonstrated changes in white matter structure as measured with diffusion tensor imaging (DTI) following treatment in individuals with a variety of psychiatric disorders, including depression (2), bipolar disorder (3), post-traumatic stress disorder (4), and obsessive compulsive disorder (5), as well as in other neurological conditions, such as cerebral palsy (6). However, unlike these other disorders, DTI has rarely been used as an outcome measure in autism clinical trials. This is despite the wealth of data supporting altered white matter structure and differential white matter developmental trajectories in autistic individuals (7–11).

While the etiology underlying differential white matter development in autism is likely heterogeneous, there is evidence suggesting that neuroinflammation may play a role, at least in a subset of individuals. Specifically, a recent prospective study linked higher levels of maternal proinflammatory markers during pregnancy to differences in cognitive development and decreased white matter integrity in frontolimbic neural circuits that are critical for social and emotional processing in the first year of life (12). Additionally, the presence of maternal IgG autoantibodies that show reactivity to fetal brain proteins have been reported in the mothers of a subset of autistic children, and this has been linked to increased brain volume of both the gray and white matter in those children (13). Similarly, in animal models of autism, neuroinflammation has been linked to both behavioral differences and increased white matter volume Reviewed in: (14–16). Taken together, these findings support investigation of novel interventions that target neuroinflammation in some autistic individuals (17) and suggest that metrics of white matter structure derived from DTI may be a particularly useful biomarker in trials for which the treatment is hypothesized to influence neuroinflammatory processes.

The current study explored white matter connectivity as a treatment efficacy biomarker in a large phase II randomized, double-blind study evaluating the safety and efficacy of cord blood treatment versus a placebo treatment in young autistic children (1). The hypothesized mechanism of action with cord blood infusion occurs via cell-to-cell signaling induced by cord blood mononuclear cells which results in downstream neuroprotective factors and reduced microglial and astrocyte activation (18). Previous research has demonstrated changes in global connectivity within white matter in children with cerebral palsy following treatment with umbilical cord blood that were also associated with clinical improvement on scales of motor abilities in the children (6). In autistic children, treatments targeting neuroinflammation have demonstrated clinical improvement across a number of measures (19). In the current study, the primary analyses from this clinical trial indicated that a single infusion of cord blood was not associated with caregiver or clinician rated improvements in social communication or other autism-related behaviors, however, secondary analyses suggested that, in the subset of autistic children who did not have co-occurring intellectual disability (ID), umbilical cord blood treatment was associated with improvements in communication based on parent report, increased clinician-rated improvement, and increased sustained attention measured via eye-tracking. In addition, changes in brain activity, specifically, increases in alpha and beta spectral power as measured with electroencephalography (EEG) were found in the subset of children without co-occurring ID (1), suggesting that there may be neurobiological changes associated with this treatment. Decreased alpha spectral power densities have been found in previous studies of autistic individuals (20).

Given the EEG evidence that cord blood treatment was associated with changes in neural activity in children without ID, the hypothesized link between cord blood treatment and decreased neuroinflammation, and the potential neuroinflammatory etiology of some white matter differences in autistic individuals, we sought to explore whether white matter structure changes were associated with cord blood treatment in the clinical trial. In particular, we focused on two metrics of white matter structure derived from DTI tractography that have previously been shown to correlate with clinical change following treatment with autologous cord blood in a separate open-label trial of *N* = 25 children (21, 22). First, we considered the number of streamlines between brain regions, a commonly employed measure of white matter structural connectivity between individual regions of interest (23). Second, we employed the Ollivier-Ricci curvature (ORC) (24), a novel geometric measure that positively correlates with changes in robustness of white matter connections among brain regions (22). Notably, we explored these metrics both within the entire group of participants, as well as in the subset of participants without co-occurring ID for which there were improvements in communication based on parent report, increased clinician-rated improvement, increased sustained attention measured via eye-tracking, and changes in EEG as reported in the original study (1).

**FIGURE 1:**
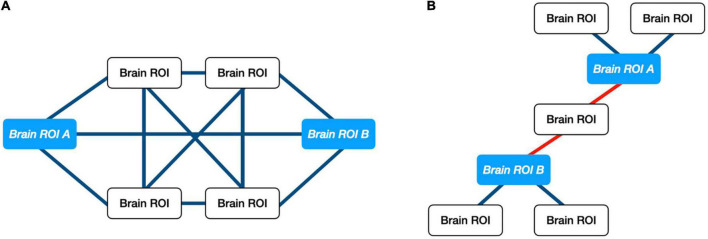
Illustration of ORC on exemplar networks. **(A)** Example of positive ORC. Multiple pathways between two brain regions implies that information between those regions may withstand small perturbations. **(B)** Example of negative ORC. A single pathway between two brain regions implies that information shared between “ROI A” and “ROI B” can be altered easily. Only a single connection would need to be perturbed.

**FIGURE 2:**
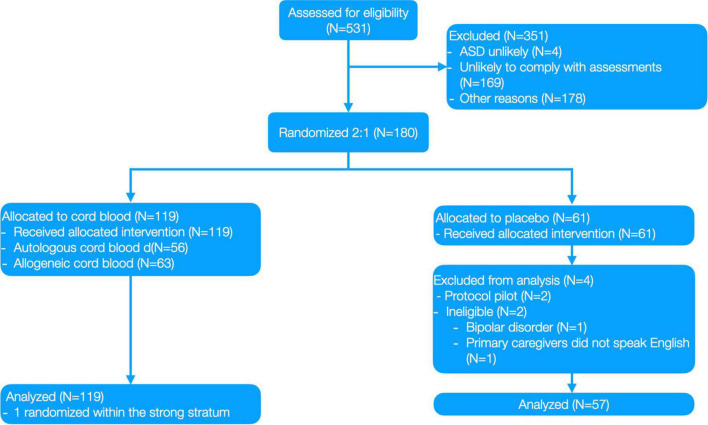
Clinical trial CONSORT diagram from Dawson, Sun (1).

Multiple studies have shown the utility of ORC, a network-science based metric of robustness in a graph, for analyzing changes in biological networks including trials involving individuals diagnosed with autism and cancer (25–30). ORC is an extension of traditional node-to-node DTI streamline analyses since it provides a measure of network robustness between a given node and all the nodes within a connected neighborhood (31). Robustness, in this context, is defined in terms of “functionality,” namely the rate at which a physical network returns to a stationary state in response to an external perturbation. Pathway robustness is characterized in terms of feedback triangles, and higher curvature (higher ORC) implies greater feedback connectivity. For control systems, feedback is a crucial method of reducing sensitivity to external disturbances and internal parameter variations. Thus, positive curvature is associated with hub-like architectures, while negative curvature is associated with tree-like architectures and therefore greater fragility. With respect to DTI data, ORC measures the relative importance of the white matter pathways between two brain regions with respect to the network architecture as a whole. For example, if two brain regions share multiple pathways, then an alteration to any given pathway will not significantly affect the amount of information that could be exchanged between the two brain regions. This relationship would be reflected by a positive ORC value. When two brain regions share only a single pathway between them, a single alteration to said pathway could negatively affect the amount of information passed between the two regions. This relationship would be reflected by a negative ORC value. Figure 1 illustrates an example of a robust and fragile connection between brain regions while Figure 2 shows the study design.

In summary, the current study builds on previous research in a separate sample of *N* = 25 children included in a phase one, open-label trial of umbilical cord blood (32) where we reported changes in both DTI streamlines (21) and ORC (22) 6 months after cord blood infusion. While the results from each of these previous papers were compelling, the lack of a control group made it impossible to determine whether or not to attribute the white matter changes to natural development or whether they were related to the treatment. To fill this gap, we applied the same analyses to the larger (*N* = 150), double-blind placebo controlled phase two trial (1) in the current manuscript. The inclusion of a placebo control group in this larger phase two trial allowed us to empirically test whether there were changes in white matter connectivity that were unique to the group of children who received treatment and therefore were unlikely to be a result of natural developmental processes. As outlined in Insel and Gogtay (33), we explored this at two levels: First, we assessed whether white matter connectivity reflected adequate treatment target engagement through measuring change in streamlines and/or ORC in the treatment as compared to the placebo group. While we did not have an a priori hypothesis about the direction of change in either the streamlines or the ORC metrics due to extensive literature documenting both underconnectivity and overconnectivity of white matter tracts in autism, we did hypothesize that changes would be centered within frontolimbic neural networks that have been shown to be both different in autistic individuals and to be impacted by maternal inflammatory markers. Then, we explored whether the degree of change in brain networks was correlated with degree of change in behavior at the individual level by examining whether the relationship between changes in DTI streamlines and ORC correlated with clinical outcomes of interest. We hypothesized that changes in the brain networks may moderate group outcomes on clinical measures.

## Materials and methods

### Study design

This paper presents a secondary analysis using data from a clinical trial (1) that was prospectively registered with ClinicalTrials.gov (NCT02847182). This was a single site, prospective, randomized, double-blind placebo-control trial of a single intravenous autologous or allogeneic cord blood infusion in children 2–7 years with autism spectrum disorder (ASD). Outcomes were assessed at baseline and 6 months after initial infusion. The IRB was approved by Duke Hospital Institutional Review Board, conducted under IND #15949, and written informed consent was obtained from all parent/legal guardians prior to study involvement. The study began September 2016; the last participant was examined for the primary outcome in August 2018. The trial protocol is available from the authors on request.

Participants were randomly assigned to Sequence A: cord blood at baseline followed by placebo infusion, or Sequence B: placebo infusion at baseline followed by cord blood infusion. Randomization was 2:1 (cord blood:placebo), stratified by age (<5 and ≥5 years), Non-verbal IQ (NVIQ; <55 vs. ≥55) and cord blood type. The randomization table was generated by RTI International (Research Triangle, Park, Durham, NC, USA). Blinded treatment codes were allocated using Medidata Rave (Medidata Solutions, New York, NY, USA). Only designated, unblinded staff (none of whom was involved in the preparation of this manuscript) were aware of the participant’s randomized treatment assignment. Complete information about the study design can be found in Figure 2 and Dawson, Sun (1).

#### Participants

A total of 180 children, 2 to 7 years of age, who met DSM-5 criteria for ASD participated. Of these children, two pilot participants and two participants who were found to be ineligible after randomization were excluded from further analysis. An additional four participants refused or withdrew prior to completing their 6-month follow-up MRI and seven participants had unusable DTI data due to excess artifacts in the data (as determined through quality checking described in the MRI preprocessing section below) at one or both time points. As a result, the final imaging dataset consisted of 165 children. Demographic data for these 165 children are shown in Table 1.

**TABLE 1:** Demographic characteristics of sample (*N* = 165).

Characteristics	Randomized groups		Cord blood subgroups	
	Treatment (*N* = 110)	Placebo (*N* = 55)	Autologous (*N* = 49)	Allogenic (*N* = 61)
Age at baseline, Mean (SD)	5.4 (1.7)	5.5 (1.6)	5.4 (1.4)	5.5 (18)
**Sex, n (%)**				
Female	19 (17%)	15 (27%)	7 (14%)	12 (20%)
Male	91 (82%)	40 (73%)	42 (86%)	49 (80%)
**Race, *n* (%)**				
White	89 (81%)	40 (73%)	38 (78%)	51 (84%)
Non-White	21 (19%)	16 (27%)	11 (22%)	10 (16%)
**Ethnicity, *n* (%)**				
Hispanic	26 (24%)	5 (9%)	5 (10%)	21 (34%)
Non-Hispanic	84 (76%)	50 (91%)	44 (90%)	40 (66%)
Non-Verbal IQ, Mean (SD)	71.2 (21.6)	73.8 (22.5)	75.1 (20.3)	68.1 (22.3)
**Non-Verbal IQ, n (%)**				
=70	59 (54%)	33 (60%)	30 (61%)	32 (52%)
≥70	51 (46%)	22 (40%)	19 (39%)	29 (48%)

Autism spectrum disorder diagnosis was based on the Autism Diagnostic Observation Schedule-2 and the Autism Diagnostic Interview, Revised (34, 35). Participants were screened for an identifiable genetic cause of autism with testing for Fragile X and chromosomal microarray. Any genetic findings were reviewed by a clinical geneticist to determine the likelihood of pathogenicity. In the setting of variants of uncertain significance, parental studies were often conducted and, if the mutation was parentally inherited and the parent was asymptomatic, it was considered non-pathogenic. Approximately 50 children were excluded due to neurogenetic findings, though that was not always their only exclusion criterion. Inclusion criteria included (1) negative genetic testing, (2) qualified cord blood (CB) unit with a minimum banked total nucleated cell dose of 2.5 × 10e7 cells/kg or ≥4/6 HLA-matched allogeneic unrelated CB unit, (3) stable on medications for ≥2 months, (4) ability to travel to study site twice, (5) English speaking, and (6) normal absolute lymphocyte count (≥1500/μL). Exclusion criteria included (1) known diagnosis of depression, bipolar disorder, schizophrenia, obsessive compulsive disorder, or Tourette syndrome; (2) known genetic syndrome or pathogenic mutation or copy number variation associated with ASD; (3) known central nervous system infection and/or HIV positivity; (4) known metabolic disorder, mitochondrial dysfunction, seizure disorder, primary immunodeficiency disorder, autoimmune cytopenias, active or prior malignancy treated with chemotherapy, significant sensory impairment, or impaired renal or liver function; (5) current or prior cell therapy, use of IV immunoglobulin or other anti-inflammatory medication (except nonsteroidal anti-inflammatory drugs), and/or immunosuppressive therapy; and (6) child unlikely to be able to complete assessments.

### Clinical assessments

Clinical improvement was assessed with the clinician rated CGI-Severity (CGI-S) and CGI-Improvement (CGI-I) scales (1, 32, 36). The CGI-S was used to rate the children’s overall level of core autism-related behavior and related functioning and support requirement at baseline and 6 months. The CGI-I was used to measure the amount of improvement or worsening of social and communicative behavior in addition to related functioning and need for supports from the time of relative to the baseline CGI-S rating. The current analyses utilized the CGI-I as the measure of clinician-rated improvement. Language abilities were assessed with the Expressive One-Word Picture Vocabulary Test-4 (EOWPVT) (37). Social, communication, and adaptive behaviors were assessed with the Vineland Adaptive Behavior Scale-Third Edition Survey Interview (VABS-3) a well-standardized caregiver interview measuring adaptive functioning, socialization, communication, daily living skills, and motor skills (38). Autism-related behaviors were assessed with the PDD-Behavior Inventory (PDD-BI) (39). The PDD-BI provides composite scores associated with Receptive/Expressive Social Communication Abilities, including both Social Approach Behavior and Expressive Language Ability domain scores, as well as an Approach/Withdrawal Problems composite score, which includes domain scores for Sensory Perceptual Approach Behaviors, Rituals/Resistance to Change Behaviors, Social Pragmatic Problems (e.g. difficulties with social awareness or making social approaches) and Semantic/Pragmatic Problems (i.e. difficulties with social communication). The PDD-BI also has an overall Composite Score, which considers both the Approach/Withdrawal Problems, as well as Receptive/Expressive Social Communication Abilities. All clinical assessments were completed at both baseline and 6 months, with the exception being the CGI-I, which was only completed at the 6-month visit.

### Magnetic resonance imaging

#### Magnetic resonance imaging acquisition

Magnetic resonance imaging scanning was conducted on a 3.0T GE MR750 whole-body 60-cm bore MRI scanner (GE Healthcare, Waukesha, WI, USA). Participants were given a participant-specific combination of dexmedetomidine, propofol, and/or midazolam for sedation prior to imaging. This was done for the purpose of increasing comfort during the infusion, which occurred immediately after the MRI scan. Diffusion-weighted images (DTI) were acquired using a 25-direction gradient encoding scheme at *b* = 800 s/mm^2^ with two non-diffusion-weighted images, an average echo time (TE) of 81.3 ms, and a repetition time (TR) of 10,000 ms. Previous research suggests that 20 directions are needed for robust estimation of fractional anisotropy and 30 directions are preferred for robust estimation of tensor orientation and mean diffusivity (40), a 25-direction gradient was selected to balance robustness of DTI tractography analyses and scan time for our pediatric participants. As such, there is the possibility that there is some error in our tensor estimates, which would influence both the streamline and the ORC data similarly, as the ORC values are directly estimated from the streamline data. An isotropic resolution of 1.5 mm^3^ was achieved using a 128 × 128 acquisition matrix in a field of view of 192 mm× 192 mm at a 1.5 mm slice thickness. T1-weighted images were obtained with an inversion-prepared three-dimensional fast spoiled-gradient-recalled (SPGR) pulse sequence with a TE of 3.19 ms, a TR of 7.14 ms, an inversion time of 400 ms, and a flip angle of 11°, at a 1 mm^3^ isotropic resolution.

#### Magnetic resonance imaging preprocessing

For each participant, structural T1 images and non-diffusion weighted images, b0, were skull stripped using the FMRIB Software Library (FSL) brain extraction tool (41, 42). The T1 image was registered to the b0 image with an affine registration created using the advanced normalization tools (ANTS) toolkit (43, 44). Quality of registration was checked manually. Region of interest (ROI) parcellation was performed by warping the dilated UNC Pediatric Brain atlas^1^ into each participant’s T1 in diffusion image space via ANTS. The parcellation results were visually inspected to confirm anatomical consistency. A total of 83 regions were defined for each participant, 41 gray matter regions in each hemisphere, and a single region encompassing the brainstem. FMRIB’s automated segmentation tool was used to calculate whole brain white matter volume for each participant at both baseline and 6-month visits (45).

Prior to running tractography analysis, diffusion-weighted images were inspected for data quality and motion corruption. Quality checking of DTI data was performed using DTIPrep, which identifies tensor images with artifacts caused by eddy-currents, head motion, bed vibration and pulsation, venetian blind artifacts, and slice-wise or gradient-wise intensity inconsistencies (46). Following this, noise level estimation and denoising based on random matrix theory was completed with the DWI denoising tool (47, 48). Eddy-current correction was then performed using FSL’s eddy correction tool and B1-field inhomogeneity correction was performed using MRTrix3 (49, 50).

#### White matter tractography and connectome construction

A standardized pipeline based on the Connectome Mapper (51) was used to analyze participant data at both baseline and 6-month visits. DTI tensors were estimated and whole-brain tractography was performed using a probabilistic algorithm utilizing the wild bootstrap (52). The reconstructed streamlines were spherical-deconvolution informed filtered to improve the quantitative accuracy of whole-brain streamlines reconstruction (53). Streamlines were then removed if they were less than 10 mm or longer than 250 mm in length. Using the UNC Pediatric Brain Atlas as a reference, streamlines were labeled by which ROIs contain their origination and termination points. Streamlines were considered orphaned and discarded if they did not begin and end in an ROI.

#### Structural connectome analysis

For graph construction, the parcellated gray matter ROIs were defined as nodes. Edges’ weights were defined as the number of valid streamlines that originate and terminate within a given pair of nodes. For each participant, edge volumes were calculated and normalized by whole-brain white matter volume at both baseline and 6-month visits. For an edge to be considered, it had to be present in each subject’s connectome at either time point 1 (baseline) or time point 2 (6 months post cord blood infusion), resulting in 158 edges.

#### Ollivier-Ricci curvature

Ollivier-Ricci curvature (24) measures the feedback connectivity of a graph network based on the comparison of the intrinsic graph distance to a distance defined via optimal transport theory between the associated probability measures given at each node (54). Formally, _κ(x,y) = 1–W_1_ (μ_x_,μ_y_)/d(x,y),_ where _κ(x,y)_ is the (edge-based) Ollivier-Ricci curvature at the nodes _x_ and _y_. Here _W_1__ denotes the Wasserstein distance, also known as the Earth Mover’s distance (EMD), between the two probability distributions. Given a number of key properties, including (weak) continuity, it is a natural distance on the space of probability distributions, with a huge associated literature (54). The distance _d_ between two nodes is the length of the shortest path connecting them (hop distance), and for a given node _x_, _μ_x__ denotes the probability distribution formed over the set of nodes in the graph in a one-step neighborhood of _x_. More precisely, we set *d*_*x*_ = ∑_*y*_ *w*_xy_ and μ_*x*_(*y*): = *w*_xy_/*d*_*x*_, where *d*_*x*_ is the sum of the weights taken over all neighbors of node *x*, and *w*_xy_ denotes the weight of an edge connecting nodes *x* and *y*. The measure μ_*x*_ may be regarded as the distribution of a one-step random walk starting from *x*, with the weight *w*_xy_ quantifying the strength of interaction between nodal components or the diffusivity across the corresponding link (edge). Further details are presented in (24, 26). The ORC is strongly connected to network robustness (31). In this context, robustness is defined as the ability of a system to adapt to dynamic changes and perturbations while still maintaining functionality. One can show that the ORC measures the robustness of the connection between two nodes while taking into account the overall connectivity of the network (22). This is a fundamental new property of this measurement. Namely, robustness is not limited to the local connectivity, but is influenced by the overall network architecture. For the longitudinal data being studied in the present work, an increase in ORC represents an increase in robustness of a given brain region, while a decrease in ORC represents an increase in fragility, once again both affected by the overall network structure.

#### Analysis plan and groups

We conducted two sets of analyses. In the first analysis, we explored changes in brain connectivity associated with cord blood infusion between the placebo and treatment groups. For this, we examined three possible combinations of treatment groups: (1) the combined autologous and allogeneic groups versus placebo, (2) autologous cord blood only versus placebo, and (3) allogeneic cord blood only versus placebo. Participants were separated based on autologous versus allogenic treatment for two reasons (1) changes in CGI-I ratings were different based on which cell type they got, as reported in Dawson et al. (1) and Gryglewski et al. (2) to account for the differences in the amount of stem cells associated with each treatment—children who received an allogenic infusion received a higher dose of cells than children who got autologous treatment (1). In addition, because of evidence that there were differential effects of treatment based on whether the child had co-occurring ID (1), we also examined differences when divided by those with NVIQ < 70 versus NVIQ ≥ 70. In the second set of analyses, we explored whether changes in brain connectivity following cord blood infusion were associated with clinical change in two ways: (1) correlating change on clinical assessments (CGI-I, EOWPVT, VABS-3, and PDD-BI) with change in the pathways for which there was a treatment effect above; and (2) looking across all brain regions, but focusing only on those clinical outcomes for which there was improvement for the subset of participants with NVIQ ≥ 70, as reported in Dawson et al. (1), namely the CGI-I and VABS-3 communication subscale.

#### Statistical analysis

Statistical analyses were written in Python using the statsmodel package (55). All models were assessed with a generalized linear model (GLM). For our analysis of change between treatment and placebo, the dependent variable for these analyses was the number of streamlines at the second time point, 6 months after infusion, the variable of interest was the group label, and the number of streamlines at baseline, nonverbal IQ, the interaction between group label and NVIQ, and age were covariates. We then repeated this analysis replacing the streamlines with ORC. In our second analyses, for each subscale and brain region pair, the relationship between changes in behavior scores between time points and changes in number of streamlines between time points was modeled with a GLM with age and NVIQ as covariates. For the CGI-I scales, the scores were dichotomized with a cutoff CGI score of three and modeled as a binomial GLM. As in the previous set of analyses, this analysis was then repeated replacing the streamlines with ORC. For a given subscale and brain measure, significance testing was corrected via the FDR Benjamini-Hochberg method (56) with an alpha value of 0.05.

## Results

**FIGURE 3:**
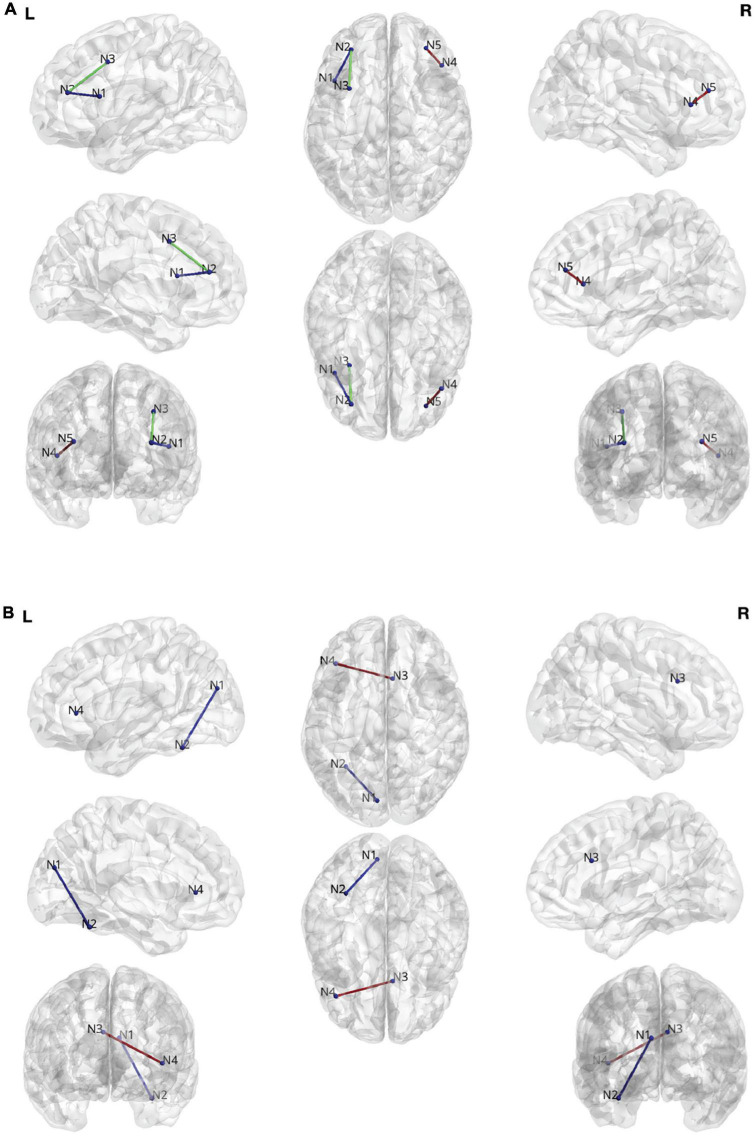
**(A)** Edges found during the streamline analysis. N1: Left Inferior Frontal Gyrus (IFG)—pars opercularis. N2: Left rostral middle frontal gyrus. N3. Left caudal middle frontal gyrus. N4. Right IFG—pars triangularis. N5. Rostral middle frontal gyrus. **(B)** Edges found during the Ollivier-Ricci curvature (ORC) analysis. N1: Left cuneus cortex. N2: Left fusiform gyrus. N3: Right caudal anterior cingulate cortex. N4: Left IFG—pars triangularis.

Figures 3A,B summarize the results comparing changes in DTI streamlines and ORC, respectively. When considering DTI streamlines as the connectivity metric of interest, there were no regions that differentiated the combined treatment group (allogenic + autologous) from the placebo group when collapsing across children with NVIQ < 70 and children with NVIQ ≥ 70. However, in the subset of children with NVIQ < 70, those who received treatment (i.e. the combined allogenic + autologous group) exhibited decreased white matter streamlines in a pathway connecting the right rostral middle frontal cortex, also known as the dorsolateral prefrontal cortex (dlPFC) to the right inferior frontal gyrus (IFG)—pars triangularis, as compared to children who received a placebo infusion (β (SE) = 0.0226 (0.006), *p* = 2.41e-4, *q* = 0.0382, Cohen’s *D* = −0.186, 95% CIs: [0.011–0.035]). Furthermore, in the subset of children with NVIQ ≥ 70 who received allogenic cord blood, we found decreased white matter streamlines between the left dlPFC and both the left IFG—pars opercularis (β (SE) = 0.0314 (0.009), *p* = 2.81e-4, *q* = 0.0222, Cohen’s *D* = −0.0262, 95% CIs:[0.014–0.048]) and the left caudal middle frontal cortex (β (SE) = −0.0470 (0.010), *p* < 3.48e-6, *q* = 5.49e-4, Cohen’s *D* = −0.0208, 95% CIs:[−0.067–−0.027])as compared to the placebo group. There were no significant differences in the number of streamlines in the subset of children with NVIQ ≥ 70 who received either just autologous cord blood, nor in the combined allogenic+autologous treatment group.

When considering ORC as the white matter connectivity metric of interest, we also found white matter connectivity changes. Specifically, in the combined treatment group (allogenic + autologous) there was a significant increase in the robustness (ORC) of the connection between the cuneus and the fusiform gyrus in the left hemisphere in the subset of children with NVIQ ≥ 70 (β (SE) = 0.106 (0.024), *p* = 7.51e-6, *q* = 0.0222, Cohen’s *D* = 0.528, 95% CIs: [0.060–0.153]). Additionally, in the subset of children who received an infusion of allogenic cord blood at baseline who also had NVIQ ≥ 70, we found increased robustness (ORC) of the connection between the right caudal anterior cingulate cortex (ACC) and the left IFG—pars triangularis (β (SE) = 0.283 (0.064), *p* = 9,64e-6, *q* = 0.0285, Cohen’s *D* = −0.156, 95% CIs: [0.158–0.408]). There were no significant differences in ORC in any of the children with IQ < 70.

We next explored whether the changes in either DTI streamlines or ORC correlated with clinical improvement on the clinician-administered measures, including the CGI-I, EOWPVT, as well as the parent-report measures, including the VABS-3 and PDD-BI. There was no correlation between the white matter changes and the clinical measures assessed. We also assessed whether there were changes in white matter anywhere in the brain that correlated with improvement on the two secondary outcomes, namely the CGI-I and the VABS-3 communication subscale, which showed improvement in the subset of children with NVIQ ≥ 70 (1). There was no correlation between improvement on these measures and either change in streamlines or ORC.

## Discussion

Intervention research in autism has historically struggled to overcome the large placebo responses that are often seen when using subjective, parent or clinician reported outcomes in clinical trials (57–61). As such, there remains a critical need for identification of complementary objective treatment efficacy and stratification biomarkers that are related to the underlying neural basis of autism. Such biomarkers would also provide a measure of target engagement as required by National Institutes of Mental Health (NIMH) sponsored clinical trials (33). To this end, the current study explored two measures of brain white matter connectivity as potential biomarkers that can be used for stratification and to assess target engagement in future clinical trials for autism: change in streamlines between brain regions and changes in network robustness as measured by ORC between brain regions. We found different patterns of white matter connectivity in predominantly frontal and temporal brain networks in autistic children who received umbilical cord blood treatment versus those who received a placebo. This suggests that DTI is a potentially objective and biologically based biomarker that maybe sensitive to biological change in clinical trials for autism.

Our first analyses compared children in the treatment as compared to a placebo-control group with respect to changes in white matter connectivity. We were interested in whether cord blood treatment was associated with brain related changes regardless of degree of change in clinical outcomes. We found that cord blood treatment was associated with changes in connectivity among a number of networks connected to the dlPFC. The dlPFC is a critical hub for supporting attention, memory, and other aspects of cognition in the brain and has been implicated as a critical region in the neurobiology of autism (62, 63). Studies employing resting state MRI have demonstrated that differential connectivity in networks involving the dlPFC correlate with social and communication abilities in autistic children (64).

In the current study, we found that cord blood treatment was associated with decreased streamline connectivity between the dlPFC and three regions: the IFG—pars opercularis and the caudal middle frontal cortex in the left hemisphere, and the IFG—pars triangularis in the right hemisphere, as compared to the placebo control group. Each of these brain regions has important roles in both social and communicative processes and have been found to be differentially engaged in autistic individuals. In particular, the left IFG—pars opercularis, otherwise known as Broca’s area, is a critical hub in the brain for language and speech production. Further, differential structure and development of the IFG—pars opercularis has been noted in autism (65). The caudal middle frontal gyrus, part of the premotor cortex, has been implicated in several roles in autism, including altered face processing and sensory hyper-responsivity (66, 67). It is also part of the mirror neuron system, implicated in social behavior in autism (68). Like the pars opercularis, the IFG—pars triangularis is also involved in language processing and plays roles in both memory and response inhibition. Studies have correlated the volume of this region with level of autism-related behaviors (69). Interestingly, while the dlPFC was consistently found across our streamline analyses, there were hemispheric differences on the dlPFC-related networks impacted depending on whether the child had co-occurring ID or not. Specifically, in children with NVIQ < 70, changes were seen in networks within the right hemisphere, whereas in the children with NVIQ ≥ 70 changes were noted in left hemisphere networks. There is precedent for differential brain findings in autistic children with and without co-occurring ID (70–72) and brain asymmetries in prefrontal regions overlapping with the pathways found in the current study have been reported in several studies (64). Of the pathways in the current study, it is of interest that the left hemisphere pathways tend to be associated with more canonical language abilities, whereas the right lateralized pathway has roles in both language and higher cognitive functions. As such, while preliminary, it is possible that the hemispheric differences found in the current study represent meaningful differences in how the brains of autistic children with and without co-occurring ID respond to treatment with cord blood. Overall, these results suggest that cord blood treatment is associated with reorganization in a network of the brain related to social and communication abilities that have previously been implicated in the neurobiology of autism.

The dlPFC findings, described above, were found in relationship to DTI streamlines. As previously mentioned, one drawback of examining DTI streamlines is that they measure connectivity between discrete pairs of brain regions without considering the role of these pairs in larger neural networks. Thus, in addition to streamlines, we also explored a novel marker of white matter brain change, ORC, which measures how robust region pairs are in the context of the broader (global) network to which they are connected. When examining ORC, we found that cord blood treatment was associated with increased robustness of the connection between the caudal ACC and the IFG—pars triangularis. The caudal ACC plays a significant role in cognitive control (73, 74), and functional activation in this region has been linked to social difficulties in autism (75, 76). Of note, there is evidence to suggest that the caudal ACC is functionally connected to the dlPFC (77). While speculative, it is possible that the ORC finding aligns with the finding of changes in dlPFC networks from the streamline analyses. Finally, there was also increased robustness in connections between the cuneus to the fusiform. This is a visual processing path, which has been implicated in face processing. Of note, changes in both the structure and the function of the fusiform in particular has been implicated across a number of studies of autism (78, 79). Using network robustness to examine changes in brain networks shows changes in brain connectivity not shown via streamlines, as shown in Farooq, Chen (26).

Of note, while there was overlap in the brain networks that were identified across analyses, we did not find any relationship between individual differences in degree of clinical change and degree of change in the white matter networks that showed differences in the treatment vs. placebo group analyses. Furthermore, following the clinical results presented in Dawson, Sun (1), we explored the relationship between individual differences in the degree of change in white matter connectivity and degree of clinical improvement as measured by the CGI-I and the VABS communication subscale in the subset of children with NVIQ ≥ 70. Importantly, clinical improvements associated with cord blood treatment for this subgroup based on IQ were part of a secondary analysis. The primary analyses for the original clinical trial indicated that cord blood is not effective for improving clinical outcomes for autistic children. In the present analyses, there were no significant associations between degree of individual-level change in either DTI streamlines or ORC and degree of improvement on either the CGI-I or the VABS communication subscale. There are several potential explanations for these findings. First, it is possible that changes in brain structural connectivity may precede behavioral changes that were not captured with the behavioral assessments in this trial. Specifically, it may be that we are capturing too broad of constructs in the summary scores from our clinical measures and that our brain-based changes may map onto more discrete clinical outcomes (e.g., social attention rather than adaptive social behavior more generally). Second, it is worth noting that there were several significant correlations prior to correcting for multiple comparisons. Thus, it is possible that we are missing relationships that are in fact there, but that we do not report to avoid type II errors. Finally, although changes in both behavior and white matter connectivity were found for the subgroup of children without ID who received cord blood treatment (specifically, allogeneic treatment was related to behavioral improvements), it is possible that degree of change in each of these domains (brain and behavior) at the individual level are not correlated, regardless of how sensitive our behavioral assays are. Nevertheless, our results suggest that DTI should be further explored as a potential target engagement endpoint in future clinical trials in autism.

A limitation of this study is that findings spanned across a number of comparisons, including children who were treated with allogenic vs. autologous cord blood, as well as those with NVIQ above and below 70. This suggests that there may be an underlying complexity to the samples, likely due to sample heterogeneity, which are driving different findings in distinct subgroups. As noted above, there is precedent for finding differential brain structure in autistic individuals with NVIQ ≥ 70 as compared to those with NVIQ < 70 (70–72). Interestingly, in the Nordahl, Dierker (70) study, there were structural differences in the IFG in autistic children with IQ < 70, which is in line with our findings. As such, it is possible that the IFG is differentially impacted in autistic children with co-occurring cognitive impairments. Future studies will need to explore whether there are ways to stratify the sample prior to an intervention, possibly including stratifying by the presence or absence of co-occurring intellectual disability, and then identify particular brain networks that better predict treatment response in that stratified sample. In addition to the heterogeneity introduced by the different IQ groups, it is possible that in the current study treatment effects were seen only in those children with underlying neuroinflammation. Unfortunately, it was not possible to test that hypothesis in the current study, but future studies exploring the relationships between neuroinflammation and white matter development in autism may help disentangle this further. Second, all of our participants were mildly sedated prior to their infusion to increase comfort and this carried over to imaging session. There is very little data on the impact of sedation on DTI measures. However, at least one study has reported transient changes in DTI measures following sedation in elderly patients (80). It is impossible to know whether sedation impacted our participants since we did not have a non-sedated baseline scan on them. However, given that all of our participants in both the treatment and the placebo groups underwent sedation prior to scanning at both time points, it is unlikely that the impact of sedation on DTI measures impacted our results. Finally, it is also of note that while there are numerous studies demonstrating white matter differences in autistic children, the pattern of differences is not always consistent. Future work will be necessary to explore whether there are subgroups of autistic children for whom there are consistent changes in white matter connectivity, which would further enhance the use of DTI as a biomarker.

## Conclusion

Overall, our results support the use of DTI to measure patterns of brain connectivity in children receiving a single infusion of umbilical cord blood that differentiate them from children in the placebo group. Changes in white matter connectivity centered on frontal and temporal brain circuits that have been previously implicated in autism. Given data suggesting that infusions of cord blood reduce neuroinflammation, such as degree of microglial activation (18), it is possible that the white matter connectivity changes we found in the treatment group represents structural changes that result from decreasing neuroinflammation, which is important given data linking neuroinflammation to larger white matter volumes in both animal models of autism and in autistic children (14, 70). That said, at the individual level, we did not find that the degree of changes in white matter connectivity were related to degree of clinical changes in the clinical trial. Thus, the results of this secondary analysis should not be interpreted as evidence for the efficacy of cord blood for improving clinical outcomes in autism. However our results suggest that future studies should consider DTI as an objective biomarker for use in clinical trials for autism, particularly those for which the mechanism of action is reduction of neuroinflammation. This is particularly important given the growing literature showing that subjective clinician observations and caregiver reports measures are easily impacted by expectancy bias (59, 61), which at times causes the placebo groups to show improvement on behavioral ratings at the same or to greater extent than that seen in the treatment groups (57, 58). Furthermore, by examining changes in network robustness, this study assessed not only the direct, physical changes in connectivity, but how the changes between two brain regions affect the robustness of the network as a whole. Because no direct connection between brain regions has yet to be considered an autism biomarker, examining network robustness as a whole may uncover patterns previously missed.

## Data availability statement

The clinical data analyzed for this study can be found in the NIMH Data Archive (NDA) repository: https://nda.nih.gov/. Imaging data will be shared upon request. Analysis code available at https://github.com/aksimhal/ORC-ACT-Analysis.

## Ethics statement

The studies involving human participants were reviewed and approved by Duke Hospital Institutional Review Board. Written informed consent to participate in this study was provided by the participants’ legal guardian/next of kin.

## Author contributions

AKS, KC, JK, AS, AT, GS, and GD: conception and design of work. AKS, KC, and LZ: analysis of data and drafting on the work. All authors were involved in the critical review of the manuscript and for important intellectual content.

## Footnotes

1
http://www.nitrc.org/projects/unc_brain_atlas/
